# Otic organoids: A model to study spiral ganglion neuron characteristics in Tmprss3-deficiency

**DOI:** 10.1016/j.isci.2025.114355

**Published:** 2025-12-05

**Authors:** André U. Deutschmann, Lucie Pifkova, Betül Findik, Moritz Klingenstein, Anton Betz, Maksim Klimiankou, Julia Skokowa, Stefan Liebau, Ellen Reisinger, Stefanie Klingenstein

**Affiliations:** 1 Gene Therapy for Hearing Impairment and Deafness, Department for Otolaryngology, Head and Neck Surgery, University Hospital Tübingen, Tübingen, Germany; 2 Department of Hematology, Oncology, Clinical Immunology, and Rheumatology, University Hospital Tübingen, Tübingen, Germany; 3 Gene and RNA Therapy Center (GRTC), University Hospital Tübingen, Tübingen, Germany; 4 Institute of Neuroanatomy and Developmental Biology (INDB), Eberhard Karls University Tübingen, Tübingen, Germany

**Keywords:** neuroscience, cell biology

## Abstract

Organoids are valuable models to study human diseases. Cochlear implants (CIs) electrically stimulate spiral ganglion neurons (SGNs) to enable severely hearing-impaired people vocal communication. However, some studies found in patients with mutations in the TMPRSS3 gene that speech comprehension with CI was lower than for other etiologies. The reduced CI performance might be associated with reduced SGN excitability, the causes for which are largely unclear. We refined a protocol for generating SGN-like cells in otic organoids from human induced pluripotent stem cells (iPSC) and confirmed their identity through marker expression and electrophysiological characterization. TMPRSS3-deficient iPSC clones developed smaller and less differentiated organoids. Moreover, TMPRSS3-deficient SGN-like cells displayed smaller currents and were less likely to exhibit action potentials, which recapitulate the expected disease phenotype. Ultimately, we seek to use this organoid model to study SGN function in human patients for enhancing our understanding and prediction of CI performance.

## Highlights

• Human otic organoids generate glutamatergic SGN-like neurons and glial cells
• SGN-like cells from otic organoids recapitulate spiral ganglion neuron subtypes
• TMPRSS3-deficient organoids show loss of SOX2/POU4F1 neuronal progenitors
• TMPRSS3 KO reduces Na^+^ currents and action potential firing in SGN-like cells

## Graphical abstract

**Figure:**
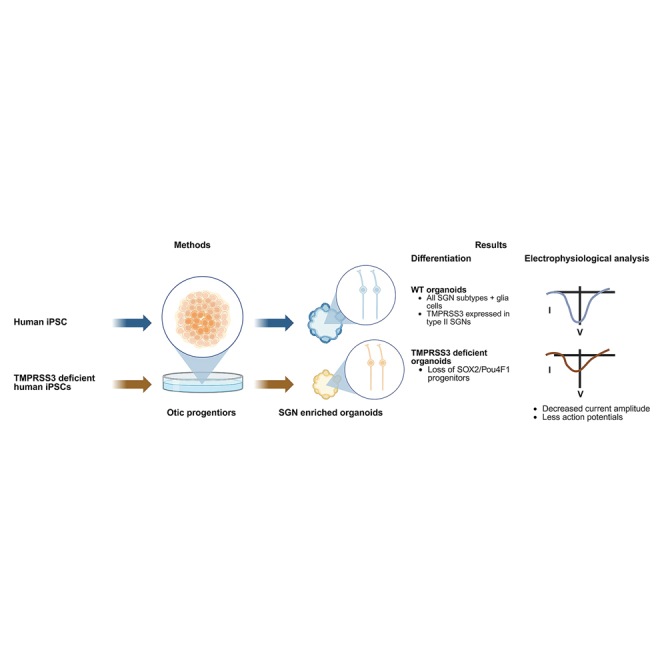

## Introduction

When sound is perceived, a mechanical stimulus reaches sensory cells in the inner ear, which transform the mechanical stimuli into electrical receptor potentials. The hair cells convey these electrical signals by synaptic transmission to spiral ganglion neurons (SGNs), the first neurons in the auditory pathway, located in the modiolus within the cochlea. These afferent neurons relay the signal, encoded as action potential trains with a sustained spike rate of 200–300 Hz, along the auditory nerve to the cochlear nucleus in the brainstem.^1^

There are two types of SGNs: type I, which make up 95% of SGNs, are exclusively innervated by inner hair cells (IHCs) and type II (5% of SGNs) contact outer hair cells (OHCs).^2^^,^^3^^,^^4^ Type I SGNs are responsible for encoding frequency and sound intensity.^5^^,^^6^ Each type I SGN is exclusively driven by one synapse from one IHC, and each IHC transmits information to 15–20 SGNs, which are encoding the same frequency but different sound intensities. To achieve the latter, type I SGNs differentiate into three different subtypes, each with different electrophysiological properties and differences in gene expression patterns.^7^^,^^8^^,^^9^^,^^10^ In rodents, this differentiation into subtypes Ia, Ib, and Ic is not completed until the fourth postnatal week, thus 2 weeks after the onset of hearing.^11^^,^^12^^,^^13^

Type II SGNs interact with several OHCs, the main function of which is amplification of the sound signal. The activation of type II SGNs triggers the medial olivocochlear reflex, which in turn suppresses cochlear amplification.^14^

Not only damage or loss of sensory cells, but also damage or loss of SGNs, e.g., due to excessive noise exposure, genetic factors, aging, or ototoxic drugs, can lead to permanent hearing impairment and deafness. Importantly, like other neurons, SGNs cannot proliferate, and while their regeneration might be achieved by endogenous cells with stem cell-like capacities, the regeneration rates are probably low since they so far have not been proven (for review see Zhang et al.^15^).

In case a loss or defect of sensory cells underlies severe or profound hearing impairment, the current standard treatment is cochlear implantation. Here, electrodes on a silicon carrier are placed into the cochlea, where they electrically stimulate the SGNs. Mainly the type I SGNs are considered to transmit this electrical signal to the subsequent auditory pathway. The more information can be transmitted from the cochlear implant (CI) to the auditory pathway, the better is the speech comprehension of the CI user. Hence, preserving type I SGNs and their electrical excitability is crucial for CI performance, and knowledge about their health would help to predict the success of a cochlear implantation. Consequently, developing a robust and functional model system is required to investigate the mechanism of hearing loss associated with SGN defects, and to develop new treatment and replacement methods for impaired or degenerated SGNs.

Variations in CI performance are associated with the age of implantation, the duration of deafness, but also the genetic etiology.^16^ In some studies, CI users with biallelic mutations in TMPRSS3 were attributed with lower than expected speech comprehension skills,^16^^,^^17^ while other studies reported some of their patients having favorable and some poor CI outcomes.^18^^,^^19^^,^^20^^,^^21^ Whether or not TMPRSS3 mutations are causative for the low performance is under debate, since other studies reported favorable results of CI implantations for these patients.^22^^,^^23^^,^^24^^,^^25^^,^^26^

Nevertheless, one study shows that electrical stimulation of the cochlea resulted in, on average, smaller neural responses in *TMPRSS3* patients, indicating a potential defect of SGNs.^16^ This led to the “spiral ganglion hypothesis,” implying that gene products with expression in SGNs or functional relevance for SGNs entail a risk for low CI performance.^27^^,^^28^

The gene *TMPRSS3* encodes the transmembrane serine protease 3, which belongs to a protein family of transmembrane serine proteases with an extracellular protease domain of unknown specificity. A mouse line with a premature stop mutation displayed a complete loss of sensory hair cells around the onset of hearing, and a severe loss of SGNs between the third and sixth months of live.^29^

Biopsies of the human inner ear containing SGNs are impossible to access without damaging the cochlea. A promising approach to overcome this obstacle is to differentiate human induced pluripotent stem cells (iPSC) into SGN-like cells.^30^ This model has already been demonstrated to be a good drug screening system,^31^ but its value for being a model system for studying SGN health remains to be elucidated.

In this study, we generated iPSCs-derived SGN-enriched organoids, and we used *TMPRSS3*-deficient organoids as disease model to evaluate this model system to study SGN health.

## Results

### Stepwise differentiation of iPSCs into SGN-enriched organoids

**Figure 1:**
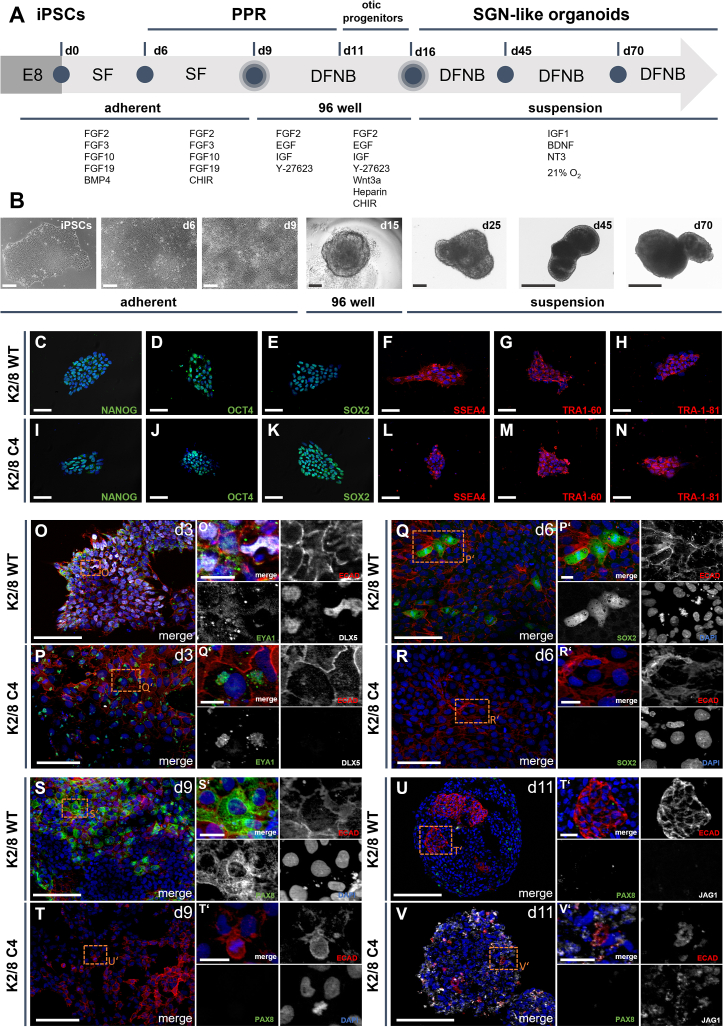
Stepwise differentiation of iPSCs into SGN-enriched organoids and early phenotypic differences in TMPRSS3-deficient clones (A) Schematic overview of the stepwise differentiation protocol. Human iPSCs were expanded in E8 medium, transferred to serum-free (SF) medium, and subsequently directed toward the otic lineage using DFNB medium under feeder-free conditions. (B) Phase-contrast images showing morphological changes during differentiation: adherent iPSC colonies (iPSCs), early aggregates (day 6 and day 9), spherical organoids (day 15), and progressively larger structures (day 25, day 45, and day 70). (C–N) Pluripotency validation of K2/8 WT (C–H) and K2/8 C4 KO (I–N) iPSCs. Nuclear markers NANOG (C, I), OCT4 (D, J), SOX2 (E, K) and surface markers SSEA4 (F, L), TRA-1-60 (G, M), TRA-1-81 (H, N) confirm robust pluripotency of all lines. (O–V) Early otic differentiation at days 3, 6, 9, and 11 in K2/8 WT (O, Q, S, U) and K2/8 C4 KO (P, R, T, V) organoids. (O, P) Day 3: K2/8 WT organoids show diffuse EYA1, ECAD^+^ epithelial boundaries, and DLX5^+^ cells, whereas K2/8 C4 organoids display perinuclear EYA1, larger ECAD^+^ cells, and no DLX5. (Q, R) Day 6: K2/8 WT retains strong nuclear SOX2 and ECAD; K2/8 C4 shows loss of SOX2^+^ progenitors and collapsed ECAD expression. (S, T) Day 9: K2/8 WT exhibits nuclear PAX8^+^ progenitors within organized ECAD^+^ epithelium; K2/8 C4 shows condensed ECAD and reduced PAX8.U, V) Day 11: K2/8 WT expresses not PAX8 and JAG1; K2/8 C4 shows early JAG1 and absent PAX8 expression. Representative images were obtained from *n* ≥ 3 organoids per time point from four independent differentiation experiments. Scale bars, 20 μm (B: iPSCs, d6, d9, C-N, O′-V′), 100 μm (B: d15, d25, O-V), and 500 μm (B: d45, d70). Nuclei were counterstained with DAPI (blue). See also Figure S2.

To generate SGN-enriched organoids, we established a stepwise differentiation protocol starting from human iPSCs (Figure 1A). Cells were initially cultured in E8 medium, followed by serum-free (SF) medium, and subsequently directed toward the otic lineage using DFNB medium under feeder-free conditions. During differentiation, we observed distinct morphological changes, including the transition from adherent iPSC colonies to progressively maturing 3D organoid structures. Representative phase-contrast images illustrate the sequential development from early aggregates (day 6 and day 9) to spherical organoids (day 15), which further increased in size and complexity until day 70 (Figure 1B).

To confirm pluripotency, all iPSC lines (two wild-type and three TMPRSS3-deficient clones) were tested for nuclear markers (NANOG, OCT4, and SOX2) and surface markers (SSEA4, TRA-1-60, and TRA-1-81). All lines displayed robust expression of these markers, demonstrating pluripotency prior to differentiation. Representative stainings for the K2/8 WT and the C4 KO line are shown in Figures 1C–1N. We next examined the early steps of otic induction by immunostaining at days 3, 6, 9, and 11. At day 3, K2/8 WT organoids displayed EYA1 expression with a diffuse cytoplasmic and partially membrane-associated distribution, together with ECAD-marked epithelial boundaries and DLX5^+^ cells, consistent with otic placodal identity (Figure 1O). In contrast, the C4 KO clone showed a markedly different EYA1 pattern, with strong perinuclear accumulation within the cytoplasm rather than diffuse distribution. ECAD was also expressed, but C4 cells appeared considerably larger than their WT counterparts, as evident from cell boundaries and the scale bar. Notably, DLX5 expression was absent in the C4 clone at this stage (Figure 1P). At day 6, WT organoids maintained strong nuclear SOX2 and ECAD expression (Figures 1Q and 1R), whereas C4 organoids exhibited a loss of SOX2^+^ progenitors and collapsed ECAD structures (Figure 1R). By day 9, WT organoids showed distinct nuclear PAX8^+^ otic progenitors alongside ECAD^+^ epithelial regions (Figure 1S). In C4 organoids, ECAD expression appeared condensed and irregular, lacking the uniform epithelial organization observed in WT. In addition, PAX8 expression was strongly reduced at this stage (Figure 1T). At day 11, WT organoids did not display detectable PAX8 or JAG1 expression in the ECAD^+^ otocyst-like regions (Figure 1U). In contrast, the C4 clone lacked PAX8 expression but already showed JAG1^+^ cells in the protruding region (Figure 1V). This reciprocal pattern indicates a marked divergence in otic patterning between WT and TMPRSS3-deficient organoids.

Together, these results show that iPSCs can be directed into otic organoids with spatially distinct ECAD^+^ otocyst-like and JAG1^+^ neuronal progenitor regions. In WT organoids, early otic induction followed a stepwise trajectory with diffuse EYA1, robust SOX2, and later onset of JAG1 expression. In contrast, TMPRSS3-deficient organoids displayed altered EYA1 localization, irregular ECAD organization, absence of DLX5, reduced SOX2 and PAX8 expression, and premature JAG1 induction. These early phenotypic differences highlight the impact of TMPRSS3 function already during initial stages of otic patterning.

### Otic patterning and neuronal differentiation in WT and TMPRSS3-deficient organoids

**Figure 2:**
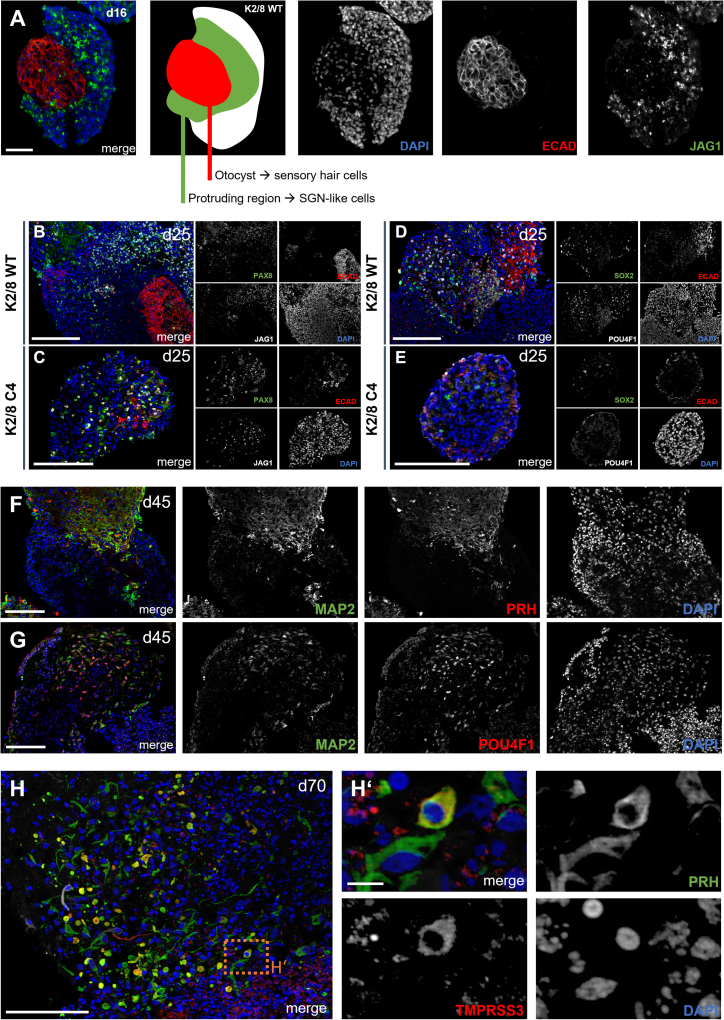
Otic patterning and neuronal differentiation in WT and TMPRSS3-deficient organoids (A) Day 16 K2/8 WT organoid showing compartmentalization into ECAD^+^ otocyst-like epithelium (red) and JAG1^+^ protruding domain (green). Schematic illustration (center) indicates spatial organization. (B, D) Day 25 K2/8 WT organoids expressing PAX8 and JAG1, together with SOX2^+^ progenitors and POU4F1^+^ neuronal precursors. (C, E) Day 25 K2/8 C4 KO organoids also expressed PAX8 and JAG1, but lacked SOX2 and POU4F1, indicating absence of progenitors and early neuronal specification. (F, G) Day 45 WT organoids display MAP2^+^ neurites co-expressing PRH (F) or POU4F1 (G). PRH is typically associated with type II SGNs, while POU4F1 marks type I SGNs, indicating the emergence of both major SGN subtypes at this stage. (H, H′) Day 70 WT organoids showing TMPRSS3 expression (red) co-localizing with PRH (green) in neuronal cell bodies. Higher magnification (H′) highlights TMPRSS3^+^/PRH^+^ neurons. Representative images were obtained from *n* ≥ 3 organoids per time point from four independent differentiation experiments. Scale bars, 100 μm (A–H) and 10 μm (H′). Nuclei were counterstained with DAPI (blue). See also Figure S3.

At day 16, WT organoids exhibited clear compartmentalization with ECAD^+^ otocyst-like regions and JAG1^+^ protruding domains, consistent with early otic patterning (Figure 2A). At day 25, both K2/8 WT and C4 organoids expressed PAX8 and JAG1, indicating progression along the otic lineage which are characteristic markers of otic progenitor cells (Figures 2B and 2C).^32^^,^^33^ However, marked differences became evident in progenitor and neuronal markers: K2/8 WT organoids showed robust SOX2^+^ progenitors and POU4F1^+^ neuronal precursors, whereas K2/8 C4 organoids lacked both SOX2 and POU4F1 expression (Figures 2D and 2E). This suggests that while otic lineage induction occurred in both conditions, TMPRSS3-deficient organoids failed to maintain SOX2^+^ progenitors and to initiate POU4F1^+^ neuronal differentiation. By day 45, WT organoids demonstrated neuronal maturation with MAP2^+^ neurites co-expressing PRH or POU4F1. PRH is typically associated with type II SGNs, while POU4F1 marks type I SGNs, indicating that both SGN subtypes emerge in WT organoids at this stage (Figures 2F and 2G).^34^^,^^35^^,^^36^ At day 70, TMPRSS3 expression was clearly detected in PRH^+^ neurons of WT organoids (Figures 2H–H′). The co-localization of TMPRSS3 with PRH confirms its presence in SGN-like cells at late stages. TMPRSS3 exhibited a slightly punctate distribution within the cytoplasm, which may indicate localization to intracellular vesicles or membrane-associated compartments. This pattern is consistent with its known function as a transmembrane serine protease typically involved in protein processing and trafficking.^29^^,^^37^ In contrast, no TMPRSS3 protein signal was observed in knockout organoids, validating both antibody specificity and the absence of TMPRSS3 protein in the KO lines.

Combined analysis of TMPRSS3 mRNA and protein at day 45 further confirmed these findings. In WT organoids, TMPRSS3 transcripts co-localized with protein in neuronal regions (Figure S3A). In contrast, all three TMPRSS3-deficient clones showed detectable TMPRSS3 mRNA but no protein signal (Figures S3B–S3D), consistent with successful knockout and confirming antibody specificity. Human kidney tissue was included as a positive control, demonstrating strong TMPRSS3 protein expression (Figure S3E).

In summary, WT organoids followed an orderly otic differentiation trajectory, maintaining SOX2^+^ progenitors and generating both type I and type II SGN-like cells. TMPRSS3 was expressed in PRH^+^ neurons with a cytoplasmic punctate distribution, consistent with its role as a transmembrane serine protease. In contrast, TMPRSS3-deficient organoids failed to sustain SOX2^+^ progenitors or initiate POU4F1^+^ neuronal differentiation. Combined mRNA and protein analyses confirmed the absence of TMPRSS3 protein in all KO clones despite residual transcript detection, validating the knockout and antibody specificity.

### Characterization of SGN-like cells and glial populations in mature organoids

**Figure 3:**
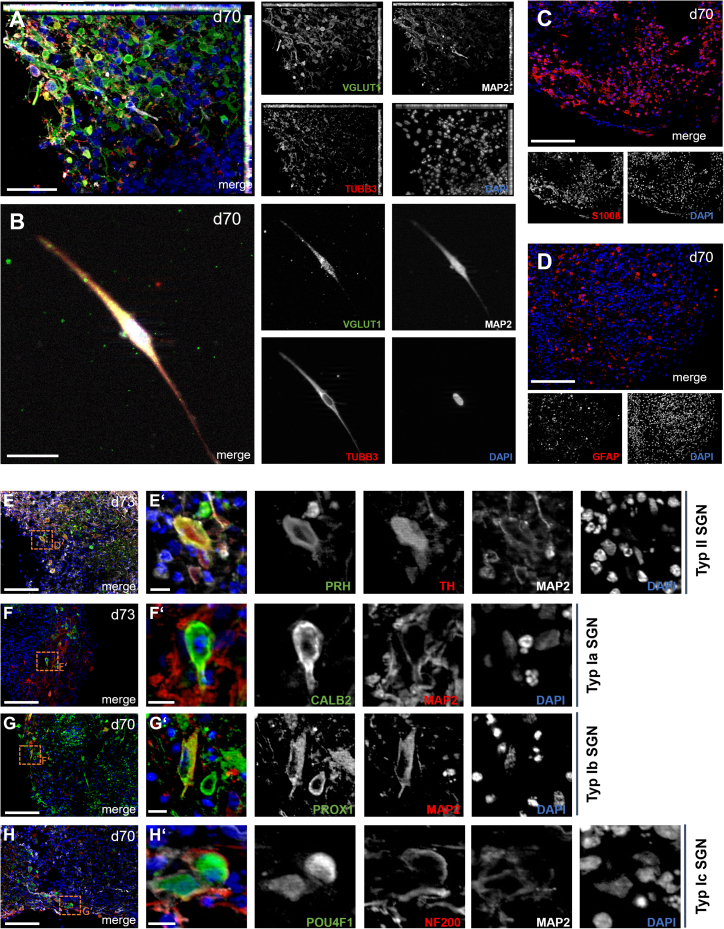
Characterization of SGN-like neurons and glial cells in day-70 otic organoids (A) Day-70 organoids stained for VGLUT1 (green), MAP2 (white), and TUBB3 (red), confirming glutamatergic identity of neurons with neuritic extensions. VGLUT1 shows punctate labeling along MAP2^+^ neurites, consistent with its synaptic localization. (B) Dissociated day-70 organoid showing a representative SGN-like cell with bipolar morphology. The soma gives rise to two opposing neurites, co-expressing MAP2 (white), TUBB3 (red), and VGLUT1 (green). (C) S100β^+^ glial cells (red), marking supporting glial populations within the organoids. (D) GFAP^+^ glial cells (red), confirming astrocytic-like glial populations in the organoids. (E–E′) PRH^+^ (green), TH^+^ (red), and MAP2^+^ (white) cells, consistent with type II SGNs. (F–F′) CALB2^+^ (green) and MAP2^+^ (white), characteristic of type Ia SGNs. (G–G′) PROX1^+^ neurons (red) with MAP2^+^ (white) morphology, consistent with type Ib SGNs. PROX1 labeling appears predominantly perinuclear with partial overlap to DAPI, consistent with its function as a transcription factor. (H–H′) POU4F1^+^ (green), NF200^+^ (red), and MAP2^+^ (white) cells, characteristic of type Ic SGNs. Representative images were obtained from *n* ≥ 3 organoids per time point from four independent differentiation experiments. Scale bars, 100 μm (C–H), 50 μm (A), 20 μm (B), 10 μm (E′–H′). Nuclei were counterstained with DAPI (blue). See also Figure S1.

At day 70, SGN-enriched organoids contained both neuronal and glial populations with features of mature cochlear tissue (Figure 3). Neurons co-expressing VGLUT1, MAP2, and TUBB3 were identified, confirming glutamatergic identity and an excitatory neuronal phenotype (Figure 3A). This finding was further corroborated by an independent staining in day-70 WT organoids, which demonstrated widespread neuronal marker expression with many cells co-expressing MAP2, TUBB3, and VGLUT1 (Figure S1A). To better visualize single-cell morphology, organoids were dissociated at day 70. High-magnification imaging revealed SGN-like cells with a characteristic bipolar morphology, showing long processes extending from opposite poles of the soma. These cells co-expressed MAP2 and TUBB3, confirming their neuronal identity, and were positive for VGLUT1, supporting their glutamatergic phenotype (Figure 3B). S100β^+^ cells were abundant and distributed throughout the organoids, indicative of Schwann cell- or satellite glia-like populations (Figure 3B).^38^^,^^39^ GFAP^+^ cells were present as well, consistent with astrocytic-like glia providing structural and trophic support (Figure 3C).^14^^,^^40^ Subtype-specific markers confirmed the heterogeneity of SGN-like neurons. PRH^+^/TH^+^ neurons, typically associated with type II SGNs, were readily detectable (Figures 3D–D′). CALB2^+^ neurons identified type Ia SGN-like cells (Figures 3E and 3F). For confirmation, additional immunostaining of type II SGN markers (PRH/NF200/MAP2) is shown in Figure S1B. PROX1^+^ neurons with MAP2^+^ morphology corresponded to type Ib SGNs (Figures 3F and 3G), while co-expression of POU4F1 and NF200 marked type Ic SGNs (Figures 3G and 3H). Together, these markers indicate that all major SGN subtypes (Ia, Ib, Ic, and II) arise within day 70 organoids.

In addition to SGN-like cells, we also detected individual MYO7A^+^ sensory cells within the organoids. At day 73, single MYO7A^+^ cells co-expressing MAP2 were identified (Figure S1C), and such cells were also present at later stages (day 182) (Figure S1D). These findings indicate that rare sensory hair cell-like cells arise within the organoids alongside the neuronal populations, which is consistent with our differentiation protocol being primarily designed to enrich for SGNs rather than to promote sensory hair cell fate.

To assess whether our differentiation protocol specifically generates SGN-like cells rather than other neuronal populations, we analyzed non-otic neural control organoids at day 70 using the same marker combinations as in SGN-enriched WT organoids (Figures 3 and S1E–S1H). While TH^+^ neurons were detected (Figure S1E), they lacked the consistent PRH/TH co-expression typical of type II SGNs. CALB2 (Figure S1F) and POU4F1 (Figure S1H) staining were negative, whereas PROX1^+^/MAP2^+^ neurons were present but displayed a distribution distinct from that observed in SGN-directed organoids (Figure S1G). None of these patterns resembled the combinatorial marker profiles characteristic of SGN subtypes. These findings confirm that the SGN-like neuronal diversity observed in SGN-enriched organoids is specific to the directed differentiation protocol and not a general feature of neuronal organoid cultures.

In summary, day 70 SGN-enriched organoids recapitulate the cellular diversity of the spiral ganglion, including glutamatergic neurons, all major SGN subtypes, and supporting glial populations. Rare MYO7A^+^ sensory cells were also detected, whereas non-otic control organoids lacked the combinatorial SGN marker patterns, underscoring the specificity of the SGN-directed differentiation protocol.

### Electrophysiological recordings indicate neuronal properties of SGN-enriched organoids

**Figure 4:**
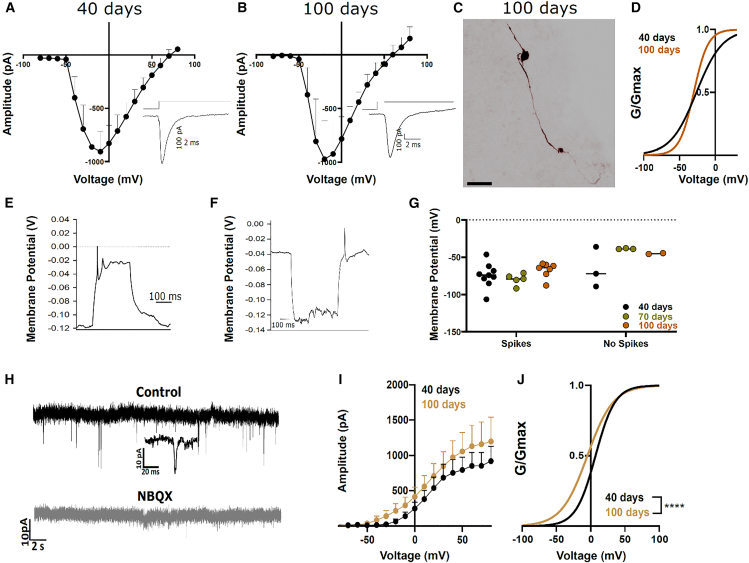
Electrophysiological recordings unravel neuron-like properties in SGN-enriched organoids (A, B) Maximum current amplitudes of voltage gated inward currents induced by depolarization steps from −80 to 80 mV from otic organoid cells after 40 days in culture (A, *n* = 13) and 100 days in culture (B, *n* = 9). Inset indicates sample traces for maximum amplitude currents. (C) Inverted fluorescent image of a patched cell, filled with biocytin and subsequently visualized by streptavidin labeling. Scale bars, 20 μm. (D) Normalized conductance for inward currents in 40 days and 100 days organoids. V½ 40 days: −26.42 mV; V½ 100 days: −30.15 mV. (E) Example trace of action potentials after a depolarizing current injection (100 days) in a rapidly adapting cell. (F) Example trace of a rebound depolarization elicited by a hyperpolarizing current injection (100 days). (G) Resting membrane potential recordings from cells with spike firing properties (total *n* = 18) and cells with no firing properties (total *n* = 8) for the indicated organoid age conditions. (H) Example trace of sEPSC recordings in an SGN like cell under control condition (black) and after the application of 10 μM NBQX (gray). Inset shows a zoomed-in view of the inward current. (I) Maximum current amplitudes for outward currents induced by depolarization steps from −80 to 80 mV after 40 days in culture (black, *n* = 9) and 100 days in culture (brown, *n* = 8). (J) Normalized conductance for 40 days (black) and 100 days (brown) inward currents. V½ 40 days: 19.69 mV; V½ 100 days: −0.98 mV, *p* < 0.001, two-way ANOVA. Data are presented as means ± standard error of the mean (SEM). Recorded cells were obtained from multiple organoids derived from independent differentiation experiments for each condition.

SGN-like cells within organoids were identified by morphological appearance and patch-clamped under whole cell configuration. We found voltage-gated inward currents in SGN-like cells with a maximum peak current amplitude of −906.8 ± 185.3 pA in 40-day-old organoids (Figure 4A; *n* = 13). In 100-day organoids, the inward current was slightly increased to a maximum peak current amplitude of −971.7 ± 357.1 pA (Figure 4A; *n* = 9). Example image of a biocytin stained 100-day-old neurons shows a bipolar shape of the recorded neuron (Figure 4C). Normalized conductance of the inward currents did not differ between 40 days and 100 days condition (Figure 4D; half maximal activation (V½ 40 days: −26.4 mV and 100 days: −30.15 mV, Boltzmann fit). In 100-day-old organoids, SGN-like cells revealed several neuronal properties: depolarizing current injections led to the generation of spikes in 18 out of 24 cells (Figure 4E; Ap thresholds: 40 days 93.7 ± 19.9 pA, 100 days 66.6 ± 10.54 pA; ns *p* = 0.25 Welch’s unpaired *t* test). Hyperpolarizing current injections induced a rebound depolarization in one cell, due to an overshoot reaction after repolarization, followed by the firing of an action potential (Figure 4F). This mechanism contributes to auditory information processing, e.g., in superior paraolivary nucleus neurons, allowing precise firing and phase-locking modulations in these cells.^5^ When comparing membrane potentials, non-spiking cells displayed a more depolarized resting membrane potential in 70- and 100-day-old organoids (*n* = 8; Figure 4G) than spiking cells (*n* = 18). SGNs are glutamatergic, and their responses can be modulated by specific compounds. During recordings, we detected spontaneous excitatory postsynaptic currents (sEPSCs) in one recorded cell. Application of the AMPA receptor inhibitor NBQX (10 μM) completely abolished these currents (Figure 4H). Potassium currents in SGNs are critical for their electrical excitability. The current-voltage relationship of outward currents in SGN-like cells showed no significant differences between 40- and 100-day conditions (Figure 4I; 40 days: *n* = 9, 100 days: *n* = 8; two-way ANOVA). In contrast, normalized conductance analysis demonstrated a pronounced leftward shift in 100-day-old organoids (Figure 4J; V½ 40 days: 19.6 mV, *n* = 9; V½ 100 days: −0.98 mV, *n* = 8; Boltzmann fit; 40 vs. 100 days: ∗∗∗∗*p* < 0.0001, two-way ANOVA).

### Inward currents in SGN-enriched organoids are TTX sensitive

**Figure 5:**
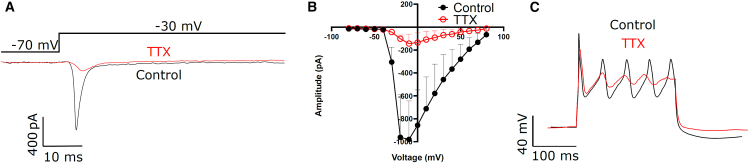
Effect of TTX on voltage-gated currents in SGN-enriched organoids (A) Traces depict currents evoked from depolarization steps from −70 mV to −30 mV under control conditions (black trace) and in the presence of TTX (1 μM, red trace). (B) Current-voltage relationship under control conditions (black line, *n* = 10) and in the presence of TTX (red line, *n* = 3). (1 μM, red trace). (C) Effect of action potential generation in the presence of TTX and under control conditions. Traces show currents after depolarizing current injections of 300 pA. Data are presented as mean ± SEM. Recorded cells were obtained from multiple organoids derived from independent differentiation experiments for each condition.

Next, we tested if the large voltage-activated currents in SGN-enriched organoids are sensitive to tetrodotoxin (TTX), indicating that these currents originate from Na_v_ channel subtypes 1.1, 1.2, 1.3, 1.6, or 1.7.^41^ Electrophysiological analysis from whole-cell recordings in acutely prepared slices of the organoids demonstrates large inward currents at depolarization steps from −70 to −30 mV for single depolarizations (Figure 5A) and in current-voltage relationship measurements (Figure 5B). Application of TTX led to a trend toward decreased current amplitude in all treated cells, suggesting that these inward currents are mainly driven by voltage-gated Na^+^ channels (Ctr; max amplitude −980.0 ± 336.6 pA, *n* = 10, and TTX 1 μM; −143.7 ± 82.9 pA, *n* = 3). The remaining current likely consists of TTX-resistant sodium channels. The application of TTX did not fully abolish action potentials in these cells (Figure 5B).

In conclusion, the SGN-like cells do express voltage-gated Na^+^-channels, as do SGNs in acutely isolated tissue.

### SGN-like cells from TMPRSS3 knockout organoids display strongly reduced voltage-gated sodium currents

**Figure 6:**
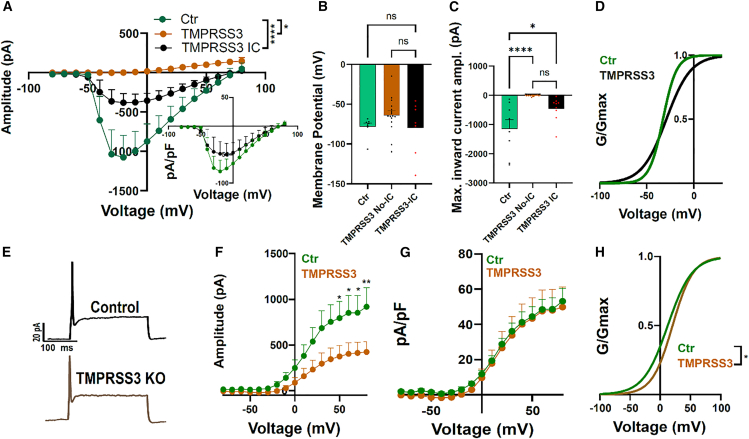
Effect of *TMPRSS3* deletion on SGN-enriched organoids (A) Current-voltage relationship indicates two different populations in *TMPRSS3*-KOs: no inward current (No-IC, *n* = 18, brown), and TMPRSS3 KOs with inward current (IC, *n* = 9 black). Control was measured in otic organoids from the original clone with intact TMPRSS3 gene (40 days old, *n* = 8, green). Inset: inward currents normalized for cell capacitance in Ctr (green) and TMPRSS3 (black). (B) Membrane potential measurements for control organoid cells (green, *n* = 7) and two populations of *TMPRSS3*-KOs (no IC, *n* = 16, brown, and IC, *n* = 7, green). (C) Maximum inward current measured in the current-voltage relationship in (A) is shown for control (green), TMPRSS3-KO cells with no inward (brown) current, and *TMPRSS3*-KO with inward currents (black). Dots represent individual cells (Ctr vs. TMPRSS3 no-IC ∗∗∗∗ <0.0001 and Ctr vs. TMPRSS3 IC *p* = 0.0126; one-way ANOVA). (D) Normalized conductance for Ctr (green) and TMPRSS3 KO (black) inward currents (V½ Ctr: −34.8 mV; V½ TMPRSS3: −32.7 mV). (E) Example traces from current-clamp recordings in control (black) and TMPRSS3 KO cells (brown) after depolarizing current injections of 350 pA. (F) Current-voltage relationship for outward currents in Ctr (green, *n* = 9) and TMPRSS3 KO (brown, *n* = 14) cells (Ctr vs. TMPRSS3: 50 mV *p* = 0.05; 60 mV = 0.26; 70 mV P = 0.03; 80 mV, *p* = 0.0082; two-way ANOVA). (I) Outward currents normalized for cell capacitance in Ctr (green, *n* = 9) and TMPRSS3 (brown, *n* = 12). (H) Normalized conductance for Ctr and TMPRSS3 KO outward currents. V½ Ctr: 12.36 mV; V½ TMPRSS3: 20.36 mV, Ctr vs. TMPRSS3, two-way ANOVA, *p* = 0.041. Data are presented as mean ± SEM. Recorded cells were obtained from multiple organoids derived from independent differentiation experiments for each condition. See also Table S1.

To investigate the functional role of TMPRSS3 in SGN-enriched organoids, we measured the electrophysiological properties of TMPRSS3-deficient SGN-like cells. The recording of voltage-gated currents from TMPRSS3 knockout (KO) cells reveals two major differences compared to control cells: most cells (18 out of 27) display no voltage-gated inward currents (No-IC; Figures 6A and 6C). However, in 9 of 27 TMPRSS3-deficient cells, inward currents (IC) could be recorded, which are significantly smaller than inward currents in control cells (Figures 6A and 6B; peak current amplitude: Ctr: −1147 ± 312.7 pA, *n* = 8; TMPRSS3-KO No-IC: −10.01 pA ± 2.1, *n* = 18; TMPRSS3-KO with IC: −462.4 ± 138.2 pA, *n* = 9; Ctr vs. TMPRSS3-KO No-IC ∗∗∗∗*p* ≤ 0.0001, Ctr vs. TMPRSS3-KO IC ∗*p* ≤ 0.01). The total membrane capacitance is directly proportional to the membrane surface cell area. When inward currents were normalized to the cell capacitance, a slight but non-significant reduction was observed in TMPRSS3-KO compared to controls (Figure 6A inset; Ctr: −79.3 ± 19.2 pA/pF, TMPRSS3-KO: −49.6 ± 19.9 pA/pF). The membrane potential was not significantly different between control, TMPRSS3-KO No-IC and TMPRSS3-KO IC cells (Figure 6C; control: −78 mV ± 4.8, *n* = 7; TMPRSS3-KO No-IC, −63.9 mV ± 5.4, *n* = 16; TMPRSS3-KO with IC, −79.4 mV ± 12.9, *n* = 8).

Normalized conductance analysis of inward currents revealed no significant differences between the two conditions (Figure 6D; Ctr V½: −32.7 mV, *n* = 9; TMPRSS3-KO: −29.1 mV, *n* = 12; Boltzmann fit). Depolarizing current injections elicited action potentials in 2 two out of 19 recorded TMPRSS3-KO SGN-like cells (Figure 6E). The current-voltage analysis revealed a significant reduction in depolarization-evoked outward responses in TMPRSS3-KO cells (Figure 6F; control max amplitude: 918.7 ± 208 pA, *n* = 9; TMPRSS3-KO: 422.8 ± 111 pA, *n* = 14; control vs. TMPRSS3-KO: +50 mV, *p* = 0.05; +60 mV, *p* = 0.026; +70 mV, *p* = 0.03; +80 mV, *p* = 0.008; two-way ANOVA with Sidák’s multiple comparisons test). However, when outward current amplitudes were normalized to cell capacitance, no significant differences were observed (Figure 6G; Ctr: 53.1 ± 7.1 pA/pF, *n* = 9; TMPRSS3-KO: 49.8 ± 11.4 pA/pF, *n* = 14), indicating that the currents scale with the plasma membrane surface area. Outward currents displayed a significant rightward shift in normalized conductance in TMPRSS3-KO cells (Figure 6H; Ctr V½: +12.36 mV, *n* = 9; TMPRSS3-KO V½: +20.36 mV, *n* = 11; Boltzmann fit, *p* = 0.04, two-way ANOVA).These data suggest that TMPRSS3 expression during the development of SGN-enriched organoids contributes to the formation and properties of excitable type I SGN-like cells, although further studies are needed to fully establish the mechanism (summarized in Table S1).

## Discussion

Our study demonstrates the successful implementation of a protocol serving to specifically enrich SGN-like cells in otic organoids from human iPSCs. During the differentiation process, the organoids developed an epithelialized structure, expressed key markers for otic tissue, and formed two distinct regions: a sensory progenitor region and a neural progenitor region, which later differentiated into different subtypes of SGN-like cells.

Although we have demonstrated that the properties of the examined cells align with the SGN criteria, we refer to them as SGN-like cells. Our study primarily focused on protein-level analyses and electrophysiological measurements, which provided detailed insights into the characteristics of these SGN-like cells. Additional RNA-level analysis could offer complementary molecular data to further substantiate their classification as SGNs.

In WT organoids, our protocol induced clear otic patterning with ECAD^+^/JAG1^+^ sensory regions next to neuronal domains. This resembles the spatial separation of sensory and neuronal progenitors in the developing otocyst and supports that the organoids follow early otic development. In TMPRSS3-deficient organoids, these structures were altered already at early stages. We observed loss of SOX2, reduced PAX8 expression, and premature JAG1 induction, indicating disturbed progenitor maintenance and sensory-neuronal balance. This suggests that TMPRSS3 may not only be important for later neuronal differentiation but also for early otic induction. Similar variability has been described in Tmprss3 mouse models, where both hair cell and neuronal defects occur, which may explain the combined sensory-neuronal phenotype in human TMPRSS3-associated hearing loss.^29^

In WT organoids, TMPRSS3 was detectable at both mRNA and protein level and co-localized with PRH^+^ neurons, suggesting an association with type II SGN-like cells. In knockout organoids, TMPRSS3 mRNA was still detectable but no protein was observed, confirming the knockout and supporting antibody specificity. Kidney tissue showed strong TMPRSS3 staining and served as positive control. The punctate cytoplasmic staining pattern in organoids fits with its role as a transmembrane serine protease. Together, these results indicate a role of TMPRSS3 in neuronal development while further work will be needed to clarify its distribution across sensory and neuronal cell types.

WT organoids developed all major SGN subtypes, including CALB2^+^ type Ia, PROX1^+^ type Ib, POU4F1^+^ type Ic, and PRH^+^/TH^+^ type II neurons. This shows that the protocol does not only yield general neurons but can reproduce SGN diversity. In addition, we observed S100β^+^ Schwann cell-like cells and GFAP^+^ astrocytic glia, suggesting that neuronal development occurs in the presence of supportive glial partners. Such interactions are important *in vivo* and may contribute to neuronal maturation in the organoids.

Non-otic control organoids did not show the same marker combinations as WT, indicating that SGN diversity is specific to the directed otic differentiation and not a general feature of neuronal organoids. Neuronal morphology also supported SGN identity: dissociated neurons showed bipolar shapes with long processes, and neurite outgrowth was seen in intact organoids. These findings argue against detection of cell bodies alone and confirm that the protocol generates neurons with characteristic projections.

Due to the experimental conditions, we were not able to unequivocally assign electrophysiological recordings to type I or type II SGN-like cells, and thus the observed electrical properties cannot be directly correlated with a specific SGN subtype.

A limitation of the otic organoid model is that it cannot fully recapitulate hair cell pathology, as only few MYO7A^+^ cells arise and they remain immature. Nevertheless, our data provide insights into the neuronal compartment and are consistent with previous reports on TMPRSS3 expression. scRNA-seq studies have indicated that TMPRSS3 is predominantly expressed in type II SGNs, while mature type I SGNs show little or no expression.^7^^,^^8^^,^^42^ In agreement with this, our parallel analysis of the rodent inner ear revealed strong Tmprss3 expression in type II SGNs but only residual or declining levels in type I SGNs. Thus, the expression pattern observed in the organoids reflects the situation in Rosenthal’s canal *in vivo*, supporting the validity of the organoid model to study TMPRSS3-related neuronal phenotypes.

Based on the results from our patch-clamp recordings, we confirmed the functional neuronal phenotype of the SGN-like cells. Key electrophysiological features characteristic of SGNs, including voltage-gated Na^+^-currents, a resting membrane potential near −70 mV, and a single or phasic firing pattern were demonstrated in SGN-like cells, indicating their functional maturation into SGNs.^43^ In particular, the presence of single and phasic firing SGNs suggests an advanced maturation stage of SGN-like cells compared to tonic and phasic firing SGNs.^44^^,^^45^^,^^46^^,^^47^ These data compare well to patch-clamp recordings of SGN-like cells in organoids from mouse iPSCs, where single-spiking, rapidly adapting and slowly adapting cells were found.^48^ Moreover, we found one cell to be excitable by glutamate via AMPA-type receptors. The fact that only one cell exhibited this behavior might point to mechanisms inhibiting synaptic contacts between SGN-like cells, since their natural drivers of excitation are hair cells, which are rare in our organoids. Our patch-clamp recordings do not allow differentiation between type I and type II SGNs. However, immunohistochemical staining confirmed the presence of both SGN subtypes. In the mature inner ear, these subtypes can be distinguished based on their biophysical properties.^49^ Nonetheless, because our study likely includes type I and type II-like SGNs at different developmental stages, the electrophysiological characteristics alone are insufficient to differentiate them here.

Our immunohistochemical data suggest that TMPRSS3 is localized in cells expressing peripherin, a marker for type II SGNs. In contrast, type I SGNs, labeled with calretinin, did neither display TMPRSS3 mRNA nor protein, which is consistent with single-cell mRNA sequencing data from acutely isolated tissue.^7^^,^^42^^,^^50^ Despite we did not find relevant expression of TMPRSS3 in type I SGNs, our data indicate an effect on the biophysical properties of SGN-like cells in the TMPRSS3-deficient SGN-enriched organoids. While our study clearly demonstrates neuronal deficits in TMPRSS3-deficient organoids, it does not fully address the sensory compartment. Our protocol is directed toward the generation of SGN-like cells, and only few inner ear sensory cells arise, which we show in the supplemental information. These remain immature and do not reach a stage where they express essential synaptic proteins such as otoferlin. Thus, otic organoids in their current state are not suitable to directly study TMPRSS3 function in hair cells. Normalization of the Na^+^ current to cell capacitance revealed that the reduced Na^+^ current amplitude observed in TMPRSS3-deficient organoids can be partly attributed to their smaller cell size. While the difference in outward current could be fully explained by reduced cell size, the trend toward decreased inward current persisted even after normalization to the cell surface area. Currently, the specific target proteins of the protease domain of TMPRSS3 remain largely unidentified, but the overall morphology of the organoids, as well as the observed electrophysiological changes, indicate a major role in otic tissue development and function. This aligns with previous findings demonstrating that TMPRSS3 knockdown leads to the degeneration of hair cells and SGNs in animal models, further underlining its importance for cell survival.^29^ Moreover, a study on mouse-derived TMPRSS3-deficient otic organoids reported a reduced number of BK channels and lower expression of genes encoding Ca^2+^-binding proteins in hair cells.^51^

Interestingly, in our TMPRSS3 knockout (KO) organoids generated using the CRISPR-Cas9 protocol, we observed a low but detectable level of TMPRSS3 mRNA expression, with slight variability across the three individual clones for review see.^52^

Importantly, TMPRSS3-deficiency causes a reduced excitability of SGN-like cells in our SGN-enriched organoids. To our knowledge, no study has directly investigated the effects of TMPRSS3 deficiency on the electrical excitability by patch-clamping of acutely isolated SGNs. In patients with bialleleic *TMPRSS3* mutations, an intracochlear electrode was employed to electrically stimulate the neurons in the ganglion spirale, which revealed a smaller auditory nerve response compared to patients with other hearing loss etiologies.^53^ Our findings would be consistent with a reduced expression of voltage-gated Na^+^-currents in SGNs developed in TMPRSS3-deficient environment, but would need to be confirmed by recordings from acutely isolated SGNs from *Tmprss3* knockout animals.

While Tmprss3 is expressed in auditory hair cells and Tmprss3 deficiency causes hair cell death between postnatal day 12 and 14 in *Tmprss3*^*Y260X*^ mutant mice^29^^,^^37^ analysis of the pathomechanism in hair cell-like cells could not be performed in our otic organoids, since here the hair-cell like cells do not reach maturity equivalent to P12–P14 mouse ears. A primary defect of hair cells is most likely underlying the observed hearing impairment, but if it is the cause of the reduced electrical excitability of SGNs remains to be determined. In mouse models, SGN cell bodies are retained for about 1 year after the loss of synaptic input.^54^^,^^55^^,^^56^ In contrast, in Tmprss3 deficient mice, half of the SGNs degenerated between P90 and P180. We thus conclude that SGN deficits are not solely due to a secondary effect of hair cell loss.

Overall, our protocol successfully generated SGN-like cells exhibiting key molecular and electrophysiological characteristics of SGNs and represents a significant step forward in generating SGN-like cells from human IPSCs. Our *TMPRSS3* knockout approach underscores the potential of these organoids to serve a model system for studying physiological properties, in particular the electrical excitability, which is most relevant for CI performance in patients. Thus, the SGN-enriched organoids are a suitable tool to understand and predict the variability in CI performance in patients with understudied or unknown etiology.

In addition to our protein-based analysis of multiple developmental stages, future work extending the current manuscript with transcriptomic profiling (e.g., scRNA-seq or bulk RNA-seq) will further strengthen the characterization of the otic differentiation trajectory.

### Limitations of the study

A key limitation of this study is the incomplete analysis of the sensory compartment. Although occasional MYO7A^+^ hair cells arise within the organoids, they remain immature and cannot be used to assess TMPRSS3 function in sensory cells. Consequently, our conclusions are restricted to the neuronal domain.

Another limitation is the lack of systematic, organoid-wide quantification of SGN enrichment or early otic progenitor markers. Instead, we focused on qualitative, protein-level analyses that highlight the most pronounced differences between wild-type and TMPRSS3-deficient organoids. As a qualitative control, we included non-otic neural organoids, which consistently lacked SGN-marker expression.

Further, we did not perform TTX pharmacology experiments in TMPRSS3-KO inner ear cells. Such experiments would help determine whether the absence of TMPRSS3 alters the balance between TTX-sensitive and TTX-resistant sodium channel activity, thereby providing further mechanistic insight into how TMPRSS3 influences excitability in inner ear cells. However, since no further cells in the preparation would have been measurable after TTX application and given the very limited number of Tmprss3 knockout organoids and available cells, this approach was not pursued.

Finally, the otic organoid model cannot fully capture the *in vivo* diversity of SGNs. While our findings align with recent data showing TMPRSS3 expression largely limited to type II SGNs,^57^ and consistent with SGN subtype analyses in Petitpré et al.,^36^ this restricts the interpretation of TMPRSS3-related pathology to neuronal subtypes that are not the major carriers of sound encoding.

The iPSC lines used in this study were derived from a single healthy 38-year-old female donor. Consequently, no sex-based comparative analyses were possible, and potential sex-specific differences in SGN development or TMPRSS3-associated phenotypes cannot be assessed in this study.

## Resource availability

### Lead contact

Further information and requests for resources and reagents should be directed to and will be fulfilled by the lead contact, Dr. Stefanie Klingenstein (stefanie.klingenstein@uni-tuebingen.de).

### Materials availability

This study did not generate new unique reagents. TMPRSS3-knockout and control iPSC lines are available from the corresponding author upon reasonable request and completion of a materials transfer agreement.

### Data and code availability

• All data reported in this paper are available from the corresponding author upon reasonable request. Raw electrophysiological trace files and representative microscopy images are available upon request. This study did not generate RNA-seq, proteomics, microarray, or other standardized high-throughput datasets requiring deposition in a public repository. No accession codes are associated with this study.
• This study did not generate any custom code.
• All materials, reagents, and experimental protocols are listed in the key resources table. *TMPRSS3*-KO clones used in this work (K2/7 A3, K2/8 C4, and K2/7 E7) are available upon reasonable request and completion of institutional MTAs.

## Acknowledgments

We thank the 10.13039/501100001659German Research Foundation (DFG) for funding this project via grant proposal #518273664 to E.R. and via the Heisenberg Program (#416097726) supporting E.R. This work was supported by the IZKF Interdisziplinäres Promotionskolleg Medizin (#2024-1-19) of the University of Tübingen, awarded to Anton Betz. We thank Sabine Conrad for technical assistance.

## Author contributions

S.K., conceptualization, investigation, formal analysis, writing – original draft preparation, writing – review and editing, validation; M.K., visualization, validation, writing – review and editing; A.B., investigation, resources; S.L., funding acquisition, supervision, writing – review and editing; B.F., investigation, formal analysis, writing – original draft preparation; M.K., investigation, formal analysis, writing – review and editing; J.S., conceptualization, project administration, supervision, methodology; E.R., funding acquisition, conceptualization, project administration, supervision, methodology, writing – review and editing; L.P., investigation, writing – review and editing; A.U.D., conceptualization, investigation, writing – review and editing.

## Declaration of interests

The authors declare no conflict of interest.

## Declaration of generative AI and AI-assisted technologies in the writing process

During the preparation of this work, the authors used ChatGPT to improve language clarity and grammar. After using this tool, the authors reviewed and edited the content as needed and take full responsibility for the final content of the publication.

## STAR★Methods

### Key resources table

**Table:** 

REAGENT or RESOURCE	SOURCE	IDENTIFIER
**Antibodies**		

CALB2	Santa Cruz	Sc-365956
CALB2	Swant	CG1
DLX5	R&D Systems	AF6710
Donkey anti-gt Alexa Fluor plus 647	Invitrogen	A32849
Donkey anti-gt Alexa Fluor647	Abcam	ab150131
Donkey anti-gt igG Alexa Fluor488	Invitrogen	A11055
Donkey anti-ms Alexa Fluor546	Thermo Fisher	A10036
Donkey anti-rb Alexa Fluor647	Jackson ImmunoResearch	711-606-152
Donkey anti-rb IgG Alexa Fluor488	Thermo Fisher	A32790
Donkey anti-rb IgG Alexa Fluor488	Jackson ImmunoResearch	711-546-152
ECAD	Life Technologies	131700
EYA1	ProteinTech	22658-1-AP
GFAP	Merck	MAB360
JAG1	Santa Cruz	Sc-390177
MAP2	Aves Lab	MAP
MYO7A	Proteus bioscience	25-6790
NANOG	Cell Signaling	4903
NF200	Sigma	N4142
OCT4	Cell Signaling	2750
PAX8	Abcam	ab97477
POU4F1	Millipore	MAB1585
PRH	Santa Cruz	Sc-377093
PROX1	Millippore	ABN278
S100ß	Abcam	ab52642
SOX2	Cell Signaling	9656S
SSEA4	Cell Signaling	4755
TH	Sigma	T2928-2ML
TMPRSS3	Abcam	AB167160
TRA-1-60	Cell Signaling	4746
TRA-1-81	Cell Signaling	4745
TUBB3	BioLegend	801201
TUBB3	BioLegend	801202
VGLUT1	Abcam	ab227805

**Biological samples**		

Human kidney tissue (formalin-fixed, post-mortem sample from body donor)	Clinical Anatomy, University of Tübingen	N/A

**Chemicals, peptides, and recombinant proteins**		

Accutase	Sigma	#A6964
Antibiotic-Antimycotic	Thermo Fisher	15240-062
ATP	Sigma	A6419
B27 Supplement	Thermo Fisher	17504-044
Biocytin	Sigma	B4261
BMP4	PeproTech	120-05ET-10UG
Bovine serum albumin (BSA)	VWR	9048-46-8
CaCl_2_	Sigma	21115
CHIR-99021	Selleck Chem	S2924
Cs-methanesulfonate	Sigma	C1426
D-Glcucose	Sigma	G 8270
DMEM/F12 + L-Glutamine + HEPES,	Thermo Fisher	31330-038
Donkey serum	Sigma	D9663
DPBS^-/-^	Thermo Fisher	14040133
EGF	PeproTech	AF-100-15-500UG
EGTA	Merck	03777
Essential8 Medium	Thermo Fisher	A1517001
FGF10	PeproTech	100-26-25UG
FGF19	Pepro Tech	100-32-25UG
FGF2	PeproTech	100-18B-50UG
FGF3	R&D Systems	1206-F3/CF
FGF8	Biotechne	423-F8
GlutaMAX	Thermo Fisher	35050-038
Growth factor-reduced Matrigel	Merck	CLS354230
GTP	Sigma	G8877
Heparin	Pepro Tech	9041-08-1
HEPES	Merck	H3375
IGF1	PeproTech	100-11-500UG
IWP2	MedChemExpress	HY-13912
KCL	Merck	P9541
L-ascorbic acid	Sigma	A8960
LDN 193189	Biotechne	6053
Low melting agarose	Carl Roth	6351.1
MgCl2	Merck	442611
Mowiol	Carl Roth	0713.1
N2 Supplement	Thermo Fisher	17502-048
Na_2_HPO_4_	Merck	S9390
NaCl	Merck	S7653
NaCl	Merck	106404
NaH_2_PO_4_	Merck	71496
NEAA	Thermo Fisher	11140-050
Normal donkey serum (NDS)	Merck Milipore	566460
NT3	Pepro Tech	450-03-10UG
O.C.T. compound	Sakura	4583
Paraformaldehyde	Carl Roth	P733.1
Skimmed milk solution	TSI GmbH	Sucofin
ß-Mercaptoethanol	Thermo Fisher	31350-010
Streptavidin 647	Invitrogen	S32357
Trisodium citrate	Carl Roth	3580.2
Triton X-100	Sigma	T8787
Triton-X	Carl Roth	3051.1
TTX	Tocris	1078
Vitronectin	Thermo Fisher	#A14700
Wnt3a	R&D Systems	5036-WN/CF
Y-27632	Selleck Chem	S1049

**Critical commercial assays**		

4D-Nucleofector X Kit L	Lonza	V4XP-3024
CytoTune-iPS Sendai Reprogramming Kit	Thermo Fisher	A16517
Neurosphere Dissociation Kit	(Miltenyi Biotec)	130-092-628
QuickExtract DNA Extraction Solution	LGC Genomics	QE09050
RNAscope Multiplex Fluorescent Reagent Kit v2	ACD Bio-Techne	323100
RNAscope Probe diluent	ACD Bio-Techne	300041
RNAscope Probe Hs-Tmprss3-C2	ACD Bio-Techne	524691-C2
TSA Vivid Fluorophore 570	ACD Bio-Techne	323272

**Experimental models: Cell lines**		

Human iPSC line K2/7 WT	This paper	N/A
Human iPSC line K2/8 WT	This paper	N/A
Human iPSC line K2/7 A3 TMPRSS3 Knockout	This paper	N/A
Human iPSC line K2/7 E7 TMPRSS3 Knockout	This paper	N/A
Human iPSC line K2/8 C4 TMPRSS3 Knockout	This paper	N/A

**Oligonucleotides**		

sgRNA TMPRSS3_ex2 (5’-CATCAAGGCCAAAAAGCGAT-3’)	Integrated DNA Technologies (IDT)	Custom synthesis
Primer TMPRSS3_ex2_F (5’-CTGAATGCGCTTTGTGTGGA-3’)	This paper	N/A
Primer TMPRSS3_ex2_R (5’-GTTGTTAGGAGCCACTCCGT-3’)	This paper	N/A

**Software and algorithms**		

DECODR v3.0	https://decodr.org	https://decodr.org/
GraphPad Prism	GraphPad Software	Version 9
ImageJ (FIJI)	NIH	https://imagej.nih.gov/ij/
PatchMaster	HEKA Electronics	PatchMaster Next
Zen Blue	Carl Zeiss Microscopy	ZEN 3.1

**Other**		

Custom media formulations (E8, SF, DFNB); see STAR Methods ‘Media formulations.	This paper	see STAR Methods ‘Media formulations’
Evos FL Imaging System	Invitrogen	N/A
HEKA Electronics	EPC 10 USB 3.0 Amplifiers Patch Clamp Amplifier	N/A
Leica Stellaris 5 Inverses Laser-Scanning Mikroskop	Leica Microsystems	N/A
Leica Vibrtome VT1000S	Leica Microsystems	N/A
Zeiss Axio Imager.M2 microscope with ApoTome.2 structured illumination module	Carl Zeiss Microscopy	N/A

### Experimental model and study participant details

#### Human iPS cell lines

Control and TMPRSS3-deficient human induced pluripotent stem cells (hiPSCs) were generated from keratinocytes isolated from plucked hair follicles of a healthy adult donor (38 years, female). The donor provided written informed consent for the use of her cells in research in accordance with the Declaration of Helsinki. Generation and handling of hiPSCs was approved by the local ethics committee of the University of Tübingen (approval number 638/2013BO1 and 424/2024BO2). The human iPSCs were cultured under standard feeder-free conditions in Essential 8 medium (E8) on vitronectin-coated culture dishes at 5% O_2_ and 5% CO_2_. Medium was replaced six days per week, and cells were passaged using Accutase according to the manufacturer’s protocol.

#### Sample size and allocation of human-derived material

All iPSC lines used in this study were derived from a single healthy adult donor (38-year-old female). Two wild-type lines (K2/7 and K2/8) and three *TMPRSS3*-knockout clones (K2/7 A3, K2/7 E7, K2/8 C4) were included. All organoid differentiation experiments were performed in multiple independent rounds, in each of which wild-type and knockout lines were processed in parallel under identical conditions. At each analyzed developmental stage, multiple organoids per clone were examined for immunostaining, and SGN-like cells for electrophysiology were recorded from several organoids across independent differentiation experiments. Allocation to WT or KO groups was determined solely by the genotype of each iPSC line.

#### Pluripotency testing of hiPSC lines

Pluripotency of all iPSC lines—two wild-type lines (K2/7 and K2/8, generated by Sendai virus–mediated reprogramming of keratinocytes) and three TMPRSS3-deficient clones (K2/7 A3, K2/7 E7, and K2/8 C4, generated by CRISPR/Cas9 genome editing)—was confirmed by immunostaining for established nuclear markers (SOX2, OCT4, NANOG) and surface markers (SSEA4, TRA-1-60, TRA-1-81). All lines showed robust expression of these markers, confirming their pluripotent state prior to differentiation.

#### Human kidney sample

Human kidney samples used as positive control for TMPRSS3 expression were obtained from body donor material provided by the Institute of Anatomy, University of Tübingen. All donors had given written informed consent during their lifetime for the use of their tissues in research and education, in accordance with the Declaration of Helsinki. The use of this material was approved by the local ethics committee of the University of Tübingen (approval number 237/2007 BO1).

### Method details

#### Media formulations

Essential (E8) medium was used for feeder-free culture of human iPSCs.

Serum-free base (SF) medium was prepared from DMEM/F12 (+L-Glutamine + HEPES) supplemented with 1% N2, 2% B27, 100 μM NEAA, 2 mM GlutaMAX, 1× Antibiotic-Antimycotic, and 0.1 mM β-mercaptoethanol.

DFNB medium was prepared from DMEM/F12 (+L-Glutamine + HEPES) supplemented with 1% N2, 2% B27, 2 mM GlutaMAX and 1× Antibiotic-Antimycotic.

#### Generation of SGN-enriched otic organoids

For organoid differentiation, protocols by Matsuoka et al. (2017) and Kurihara et al. (2022) were adapted. On day −1, iPSCs were dissociated with Accutase and seeded at 1 × 10ˆ5 cells per well in E8 medium supplemented with 10 μM Y-27632. Y-27632 was removed after 24 h, and differentiation was initiated on day 0 by switching to serum-free (SF) medium. From days 3–5, SF medium was supplemented with FGF2, FGF3, FGF10, FGF19, and BMP4. Between days 6–8, BMP4 was replaced by CHIR-99021. On day 9, cells were dissociated again with Accutase and transferred to low-attachment 96-well plates at 5 × 10ˆ3 cells per well in DFNB medium. At this stage, cultures were supplemented with FGF2, EGF, IGF1, and Y-27632 for two days, followed by Wnt3a, heparin, and CHIR-99021 from day 11–15. Medium was changed daily. From day 16, organoids were transferred into non-adherent suspension flasks (four organoids per well) and maintained under 21% O_2_ and 5% CO_2_ in DFNB medium supplemented with IGF1, BDNF, and NT3. The medium was replaced three times per week. Six independent differentiation experiments were performed, using two Sendai virus–reprogrammed wild-type iPSC lines (K2/7 and K2/8) and three TMPRSS3-knockout clones (K2/7 A3, K2/7 E7, and K2/8 C4).

#### Dissociation and adherent culture of organoid-derived cells

Organoids at day ∼70 of differentiation were dissociated into single cells using the Neurosphere Dissociation Kit (Miltenyi Biotec) according to the manufacturer’s protocol. Approximately 30,000 cells were plated per glass coverslip (Ø 10 cm dish) that had been pre-coated with growth factor–reduced Matrigel (diluted 1:100 in DMEM/F12) for 1 h at room temperature. Cells were cultured in DFNB medium supplemented as described above, with the addition of 10 μM Y-27632 for the first 24 h. Thereafter, the inhibitor was removed and cells were maintained in DFNB medium for a total of 72 h before fixation for immunocytochemistry.

#### Generation of non-otic neural control organoids

Non-otic neural control organoids were generated in parallel from the same iPSC clones and share the early differentiation trajectory with otic organoids, including induction of the preplacodal ectoderm. At the stage of placodal specification, cultures were directed away from an otic fate by adding alternative growth factors (BMP4, IWP2, LDN 193189, FGF8) known to promote non-otic sensory lineages. The resulting organoids reproducibly developed into neural sensory structures that did not express SGN-specific markers. These non-otic neural control organoids served as a negative control in the present study. A detailed differentiation protocol will be described in a forthcoming publication.

#### Immunostaining (organoids and adherent cells)

Organoids were fixed for 15 min at room temperature in 4% paraformaldehyde containing 10% sucrose, washed in DPBS^-/^, embedded in O.C.T. compound, and cryosectioned at 14 μm (Microm HM560; specimen −21 °C, blade −20 °C). Sections were circled with a hydrophobic pen and passed through an ethanol series (70%–95%–100%–95%–70%; 30 s each), rehydrated in DPBS^-/^. For blocking, samples were incubated for 45 min at room temperature in DPBS^-^/^-^ containing 10% normal donkey serum, 5% bovine serum albumin, 4% skimmed milk, and 0.1% Triton X-100. Primary antibodies were diluted in the same blocking solution and incubated overnight at 4 °C. Primary antibodies (see key resources table) were diluted in blocking buffer and incubated overnight at 4 °C. The next day, sections were washed 3×5 min in DPBS^-/^ and incubated with Alexa-conjugated secondary antibodies for 1 h at room temperature protected from light, washed (DPBS^-/-^, water), and mounted with Mowiol. Immunostaining of adherent, dissociated cells followed the same protocol except fixation was with 4% paraformaldehyde (no sucrose) and the ethanol series was omitted. Imaging parameters are described under “microscopy, image acquisition, and processing.”

#### Combined RNAscope and immunostaining

For RNAscope, organoids were fixed and cryosectioned as described for immunostaining. Sections were outlined with a PAP pen, air-dried 5 min, and processed using the RNAscope Multiplex Fluorescent Reagent Kit v2 (see key resources table). All hybridization and amplification steps were performed at 40 °C in a humidified oven. Sections were rehydrated in DPBS^-/-^, post-fixed in 4% paraformaldehyde for 30 min, treated with RNAscope Hydrogen Peroxide for 10 min at room temperature, and incubated with Protease III for 10 min. The RNAscope probe Hs-Tmprss3-C2 was diluted 1:50 in probe diluent and hybridized for 2 h at 40 °C. After washes in RNAscope buffer, sections were stored overnight in 5× SSC buffer at room temperature. Amplification steps Amp 1 and Amp 2 were performed for 30 min each, Amp 3 for 15 min, followed by incubation with HRP-C2 for 15 min, TSA Vivid Fluorophore 570 (1:1500 in TSA buffer), and HRP blocker. For combined detection of protein, sections were blocked with DSDB buffer (17 % Donkey serum, 0.3 % Triton X-100, 20 mM Phosphate buffer pH 7.4, and 0.45 M NaCl in H_2_O for 1 h at room temperature, incubated with primary antibodies overnight at 4 °C, and washed three times. Alexa-conjugated secondary antibodies were applied for 1 h at room temperature. After additional washes, sections were counterstained with RNAscope DAPI and mounted with Mowiol.

#### Microscopy, image acquisition, and processing

Brightfield images were acquired using an Evos FL Imaging System. Immunofluorescence images were obtained on a Zeiss Axio Imager.M2 microscope equipped with an ApoTome.2 structured illumination module. Objectives used included a Plan-APOCHROMAT 20×/0.8 and a Plan-APOCHROMAT 63×/1.4 Oil M27. For each field of view, Z-stacks were acquired to capture optical sections through the organoid tissue. Image stacks were processed with ZEN Blue software (Zeiss). Maximum Intensity Projections were generated to combine focal planes into single representative images. Brightness and contrast adjustments were applied uniformly across the dataset without altering original image content.

#### Organoid slice preparation for electrophysiology

Organoids were collected using a cut 200 μL pipette tip and embedded in 4% low-melting agarose dissolved in extracellular recording solution containing; in mM: NaCl 145, KCl 3, CaCl_2_ 2, MgCl_2_ 1.3, HEPES 10, L-ascorbic acid 0.4, Glucose 10; pH adjusted to 7.3; osmolarity 300–305 mOsm). Up to 2–3 organoids were placed into a single agarose block before solidification. Tissue slices of 150 μm thickness were cut using a vibratome (VT1000S, Leica Microsystems) and transferred into extracellular solution. Slices were allowed to equilibrate for 15–30 min before being transferred to the recording chamber, which was continuously perfused with Phys-solution at a rate of 1 ml/min.

#### Whole-cell recordings

Recording pipettes were pulled from borosilicate glass capillaries (World Precision Instruments) to a resistance of 3–6 MΩ, coated with Sylgard, and filled with intracellular solution containing (in mM): KCl 135, CaCl_2_ 0.1, MgCl_2_ 3.5, HEPES 5, EGTA 5, ATP 2.5, and GTP 1 (pH 7.3). Recordings were performed with an EPC-10 amplifier (HEKA Electronics) controlled by PatchMaster Next software. Currents were low-pass filtered at 5 kHz and sampled at 20 kHz. Access resistance and leak currents were continuously monitored; recordings were discarded if either changed by >25% during acquisition. Slices were visualized using a Leica microscope equipped with a 40× water immersion objective. SGN-like cells were identified based on morphology. After establishing a gigaohm seal, whole-cell configuration was obtained at a holding potential of –70 mV, with correction for liquid junction potential. Voltage-clamp recordings used depolarization steps from –80 mV to +80 mV in 10 mV increments. Resting membrane potential and action potentials were recorded under current-clamp conditions. For action potential recordings, current was injected in steps from –300 pA to +500 pA in 50 pA increments. *sEPSC:* sEPSC were recorded using the following intracellular solution (in mM): 120 Cs-methanesulfonate, 3 NaCl, 10 HEPES, 5 EGTA, 2 ATP, 0.3 GTP, 1 MgCl_2_ (pH 7,3: 290 mOsm kg^-1^). Whole-cell recordings were initiated 2 min after achieving stable whole-cell access to allow an exchange between the pipette solution and the intracellular fluid. All recordings were performed at room temperature (22–25°C).

#### Labeling of patched cells

To identify patched neurons after electrophysiological recordings, biocytin (0.1%) was included in the internal pipette solution. The pipette was attached to the cell for at least 30 min to allow the diffusion of biocytin through the cell. Following recordings, organoids were fixed with 4% FA overnight. The cells were blocked with DSDB for 8 h and incubated with streptavidin-conjugated Alexa Fluor 647 overnight to visualize the biocytin-filled cells.

#### Data analysis for electrophysiology

Electrophysiological data were analyzed using PatchMaster Next (HEKA Electronics) and GraphPad Prism (GraphPad Software, v9). Statistical analyses and sample sizes (n, number of cells) are reported in the figure legends. Differences between groups were tested using unpaired two-tailed Student’s t-tests and 1- way and 2-way ANOVA. Significance was defined as p ≤ 0.05. Results are annotated as follows: ns, p > 0.05; ∗ p ≤ 0.05; ∗∗ p ≤ 0.01; ∗∗∗ p ≤ 0.001.

#### Generation of TMPRSS3 knockout iPSCs

Two wild-type iPSC lines (K2/7 and K2/8), reprogrammed from plucked keratinocytes of healthy donors using Sendai virus, were cultured on Geltrex-coated plates in StemFlex medium supplemented with Antibiotic-Antimycotic (see key resources table). TMPRSS3 knockout was generated by CRISPR/Cas9 genome editing. A synthetic sgRNA targeting exon 2 of TMPRSS3 (5′-CATCAAGGCCAAAAAGCGAT-3′) was complexed with Cas9 protein (15 μg) to form ribonucleoprotein (RNP) complexes by incubation for 25 min at room temperature. RNPs were delivered by nucleofection using the 4D-Nucleofector system (Lonza) with program CA-137 and the X Kit L. Editing efficiency was assessed 72 h post-nucleofection. Genomic DNA was extracted with QuickExtract solution, and the target locus was amplified by PCR using TMPRSS3_ex2_F (5′-CTGAATGCGCTTTGTGTGGA-3′) and TMPRSS3_ex2_R (5′-GTTGTTAGGAGCCACTCCGT-3′). Amplicons were analyzed by Sanger sequencing and deconvoluted using DECODR software. The frequency of frameshift indels in the bulk population was 31% (K2/7) and 36% (K2/8). For clonal selection, edited iPSCs were seeded at low density, individual colonies were manually picked under a microscope and expanded in 96-well plates. After genotyping, three homozygous clones were selected: K2/7 A3 (29 bp deletion), K2/7 E7 (4 bp deletion), and K2/8 C4 (7 bp insertion) (see Figure S2). These, together with the parental wild-type lines, were used for organoid differentiation experiments.

### Quantification and statistical analysis

Quantitative data are presented as mean ± SEM. Statistical analyses were performed using GraphPad Prism software. Normal distribution was assumed based on data structure and residual analysis; however, sample sizes were limited. Differences between groups were tested using unpaired two-tailed Student’s t-tests, with a significance threshold set at p ≤ 0.05. The number of biological replicates (n) is specified in the respective figure legends. Electrophysiological traces were analyzed using PatchMaster Next, and quantification of immunofluorescence was performed manually in FIJI/ImageJ where indicated. Exact p-values are indicated in the figure legends whenever asterisks are used (∗p < 0.05, ∗∗p < 0.01, ∗∗∗p < 0.001).
