# Supplementary material for: Otic organoids: A model to study spiral ganglion neuron characteristics in Tmprss3-deficiency
Source: iScience. 2025 Dec 5;29(1):114355. doi: 10.1016/j.isci.2025.114355 (PMC12775872; doi:10.1016/j.isci.2025.114355)
Supplement: Document S1. Figures S1–S3 and Table S1 [file mmc1.pdf]

iScience, Volume *29*

Supplemental information

Otic organoids: A model to study spiral ganglion neuron characteristics in Tmprss3-deficiency

André U. Deutschmann, Lucie Pifkova, Betül Findik, Moritz Klingenstein, Anton Betz, Maksim Klimiankou, Julia Skokowa, Stefan Liebau, Ellen Reisinger, and Stefanie Klingenstein

SUPPLEMENTAL INFORMATION TITLES AND LEGENDS

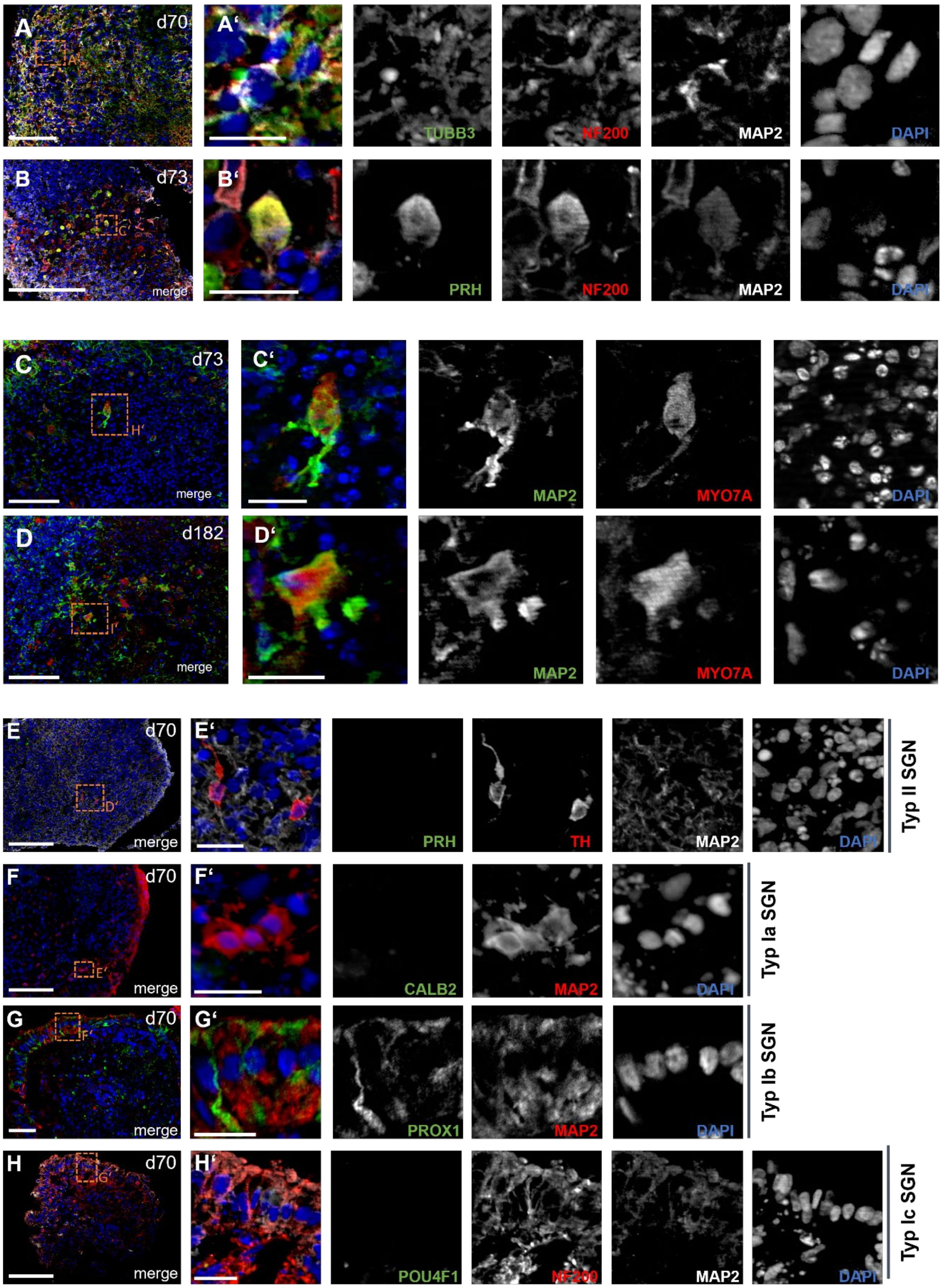

Supplemental Figure S1. Related to Figure 3. Additional characterization of neuronal and sensory cell types in wildtype otic and non-otic organoids

A) Day-70 otic organoid showing widespread neuronal marker expression. Many cells co-express MAP2 (white), TUBB3 (red), and VGLUT1 (green), confirming neuronal identity and glutamatergic phenotype. B) Day-73 otic organoid stained for PRH (green), NF200 (red) and MAP2 (white), confirming the presence of type II SGN-like neurons. C) Day-73 otic organoid showing individual MYO7A^{+} sensory cells (red) co-expressing MAP2 (green). D) Day-182 otic organoid with MYO7A^{+} sensory cells (red) at late stages, co-expressing MAP2 (green). E–H) Non-otic organoids at day 70 stained with the same SGN subtype markers as otic organoids. E) TH^{+} cells were present but lacked the consistent PRH/TH co-expression characteristic of type II SGNs. F) CALB2 staining was negative. G) PROX1^{+}/MAP2^{+} neurons were observed but their distribution and localization differed from the patterns seen in SGN-directed organoids. (H) POU4F1 staining was negative. None of these patterns resembled the SGN subtype profiles observed in SGN-directed organoids. Representative images were obtained from n ≥ 3 organoids per time point from four independent differentiation experiments. Scale bars: 20 µm (A'-H), 50 µm (E-H), 100 µm (A-D). Nuclei counterstained with DAPI (blue).

Genotyping results of iPSC clone K2/7 A3

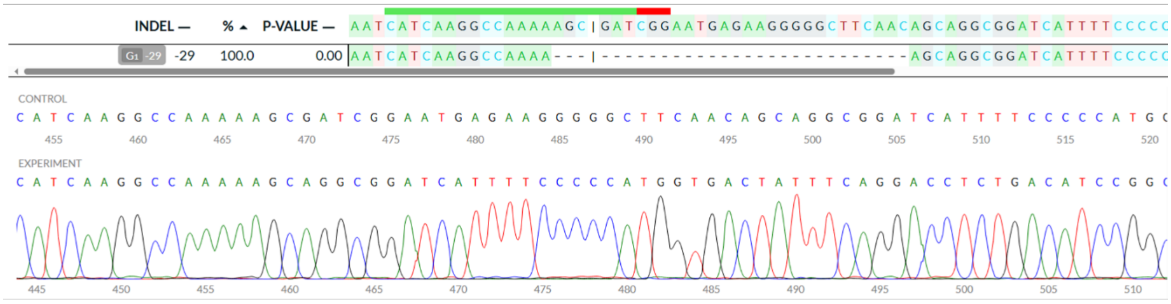

Genotyping results of iPSC clone K2/8 C4

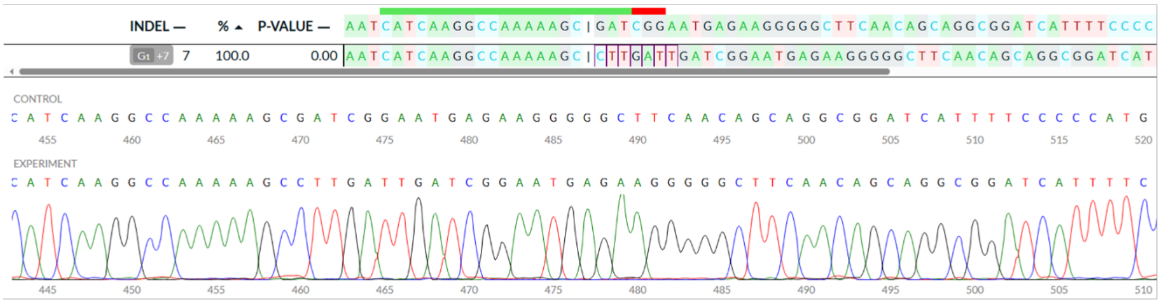

Genotyping results of iPSC clone K2/7 E7

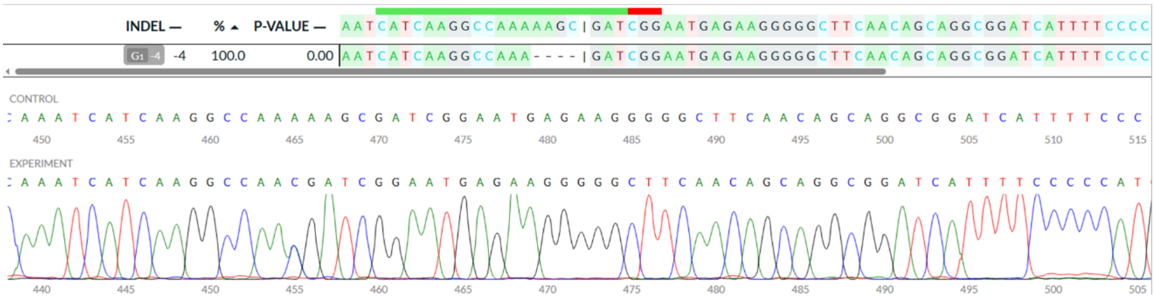

Supplemental Figure S2. Related to Figure 1. Genotyping results of single-cell derived iPSC clones

Indels percentages were calculated using results of Sanger sequencing and Deconvolution of Complex DNA Repair, DECODR v.3.0 tool. The PAM sequence is marked with a red line; the sgRNA sequence is indicated by a green line.

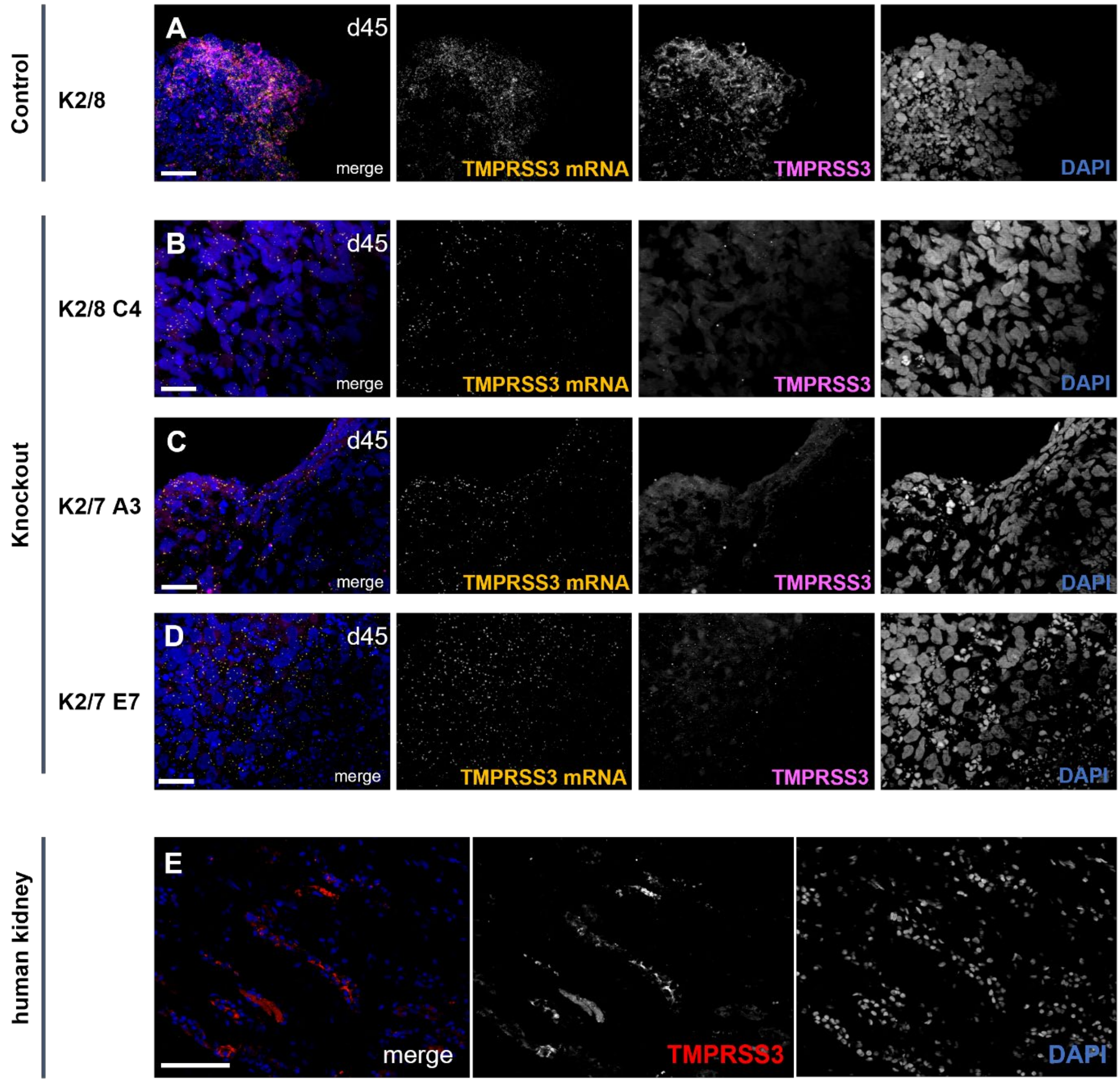

Supplemental Figure S3. Related to Figure 2. TMPRSS3 mRNA and protein detection in day-45 organoids

A) K2/8 WT organoid showing TMPRSS3 mRNA (yellow) and TMPRSS3 protein (magenta) expression, with co-localization in neuronal regions. B–D) Representative knockout organoids (K2/8 C4, K2/7 A3, K2/7 E7) displaying detectable TMPRSS3 mRNA but absence of TMPRSS3 protein signal, consistent with successful knockout and confirming antibody specificity. E) Human kidney tissue stained as antibody positive control, exhibiting strong TMPRSS3 protein expression. Representative images were obtained from n ≥ 3 organoids per time point from four independent differentiation experiments. Scale bars: 20 μm (A-D), 100 μm (E). Nuclei counterstained with DAPI (blue).

Supplemental Table S1. Related to Figure 6.

Summary of electrophysiological data, including maximum current amplitude, V½, and normalized currents for the indicated conditions.

Condition	Inward Amplitude (pA)	Outward Amplitude (pA)	Inward V½ (mV)	Outward V½ (mV)	Norm. for Capacitance inward currents (pA/pF)	Norm. for Capacitance outward currents (pA/pF)
40 days	-906.8 ± 185.3	918.7 ± 209	-26.4	19.6		
100 days	-971.7 ± 357.1	1197 ± 341	-30.1	-0.98		
TMPRSS Ctr	-1147 ± 312.7	918.7 ± 208	-32.7	12.36	-79.3 ± 19.2	53.1 ± 7.1
TMPRSS3 KO	-462.4 ± 138.2	422.8 ± 111	-29.1	20.36	-49.6 ± 19.9	49.8 ± 11.4
